# Booster vaccination against SARS-CoV-2 induces potent immune responses in people with HIV

**DOI:** 10.1093/cid/ciac796

**Published:** 2022-10-05

**Authors:** Sarah Fidler, Julie Fox, Timothy Tipoe, Stephanie Longet, Tom Tipton, Movin Abeywickrema, Sandra Adele, Jasmini Alagaratnam, Mohammad Ali, Parvinder K Aley, Suhail Aslam, Anbhu Balasubramanian, Anna Bara, Tanveer Bawa, Anthony Brown, Helen Brown, Federica Cappuccini, Sophie Davies, Jamie Fowler, Leila Godfrey, Anna L Goodman, Kathrine Hilario, Carl Philipp Hackstein, Moncy Mathew, Yama F Mujadidi, Alice Packham, Claire Petersen, Emma Plested, Katrina M Pollock, Maheshi N Ramasamy, Hannah Robinson, Nicola Robinson, Patpong Rongkard, Helen Sanders, Teona Serafimova, Niamh Spence, Anele Waters, Danielle Woods, Panagiota Zacharopoulou, Eleanor Barnes, Susanna Dunachie, Philip Goulder, Paul Klenerman, Alan Winston, Adrian V S Hill, Sarah C Gilbert, Miles Carroll, Andrew J Pollard, Teresa Lambe, Ane Ogbe, John Frater

**Affiliations:** Department of Infectious Disease, Faculty of Medicine, Imperial College London, London, UK; Department of HIV Medicine, St Mary's Hospital, Imperial College Healthcare NHS Trust, London, UK; NIHR Imperial Clinical Research Facility and NIHR Imperial Biomedical Research Centre, London, UK; NIHR Guy’s and St Thomas’ Biomedical Research Centre; Department of Infection, Harrison Wing and NIHR Clinical Research Facility, Guys and St Thomas’ NHS Trust, London, UK; Peter Medawar Building for Pathogen Research, Nuffield Dept of Clinical Medicine, University of Oxford, Oxford, UK; Wellcome Centre for Human Genetics, Nuffield Department of Medicine, University of Oxford, Oxford, UK; Wellcome Centre for Human Genetics, Nuffield Department of Medicine, University of Oxford, Oxford, UK; Department of Infection, Harrison Wing and NIHR Clinical Research Facility, Guys and St Thomas’ NHS Trust, London, UK; Peter Medawar Building for Pathogen Research, Nuffield Dept of Clinical Medicine, University of Oxford, Oxford, UK; Department of Infectious Disease, Faculty of Medicine, Imperial College London, London, UK; Department of HIV Medicine, St Mary's Hospital, Imperial College Healthcare NHS Trust, London, UK; Peter Medawar Building for Pathogen Research, Nuffield Dept of Clinical Medicine, University of Oxford, Oxford, UK; Oxford Vaccine Group, Department of Paediatrics, University of Oxford, Oxford, UK; Department of Infection, Harrison Wing and NIHR Clinical Research Facility, Guys and St Thomas’ NHS Trust, London, UK; Department of Infection, Harrison Wing and NIHR Clinical Research Facility, Guys and St Thomas’ NHS Trust, London, UK; NIHR Imperial Clinical Research Facility and NIHR Imperial Biomedical Research Centre, London, UK; Department of Infection, Harrison Wing and NIHR Clinical Research Facility, Guys and St Thomas’ NHS Trust, London, UK; Peter Medawar Building for Pathogen Research, Nuffield Dept of Clinical Medicine, University of Oxford, Oxford, UK; Peter Medawar Building for Pathogen Research, Nuffield Dept of Clinical Medicine, University of Oxford, Oxford, UK; The Jenner Institute, Nuffield Department of Medicine, University of Oxford, Oxford, UK; The Jenner Institute, Nuffield Department of Medicine, University of Oxford, Oxford, UK; The Jenner Institute, Nuffield Department of Medicine, University of Oxford, Oxford, UK; The Jenner Institute, Nuffield Department of Medicine, University of Oxford, Oxford, UK; Department of Infection, Harrison Wing and NIHR Clinical Research Facility, Guys and St Thomas’ NHS Trust, London, UK; Medical Research Council Clinical Trials Unit, University College London, London, UK; Department of Infection, Harrison Wing and NIHR Clinical Research Facility, Guys and St Thomas’ NHS Trust, London, UK; Peter Medawar Building for Pathogen Research, Nuffield Dept of Clinical Medicine, University of Oxford, Oxford, UK; Department of Infection, Harrison Wing and NIHR Clinical Research Facility, Guys and St Thomas’ NHS Trust, London, UK; Oxford Vaccine Group, Department of Paediatrics, University of Oxford, Oxford, UK; Department of Infection, Harrison Wing and NIHR Clinical Research Facility, Guys and St Thomas’ NHS Trust, London, UK; Department of Infectious Disease, Faculty of Medicine, Imperial College London, London, UK; Department of HIV Medicine, St Mary's Hospital, Imperial College Healthcare NHS Trust, London, UK; Oxford Vaccine Group, Department of Paediatrics, University of Oxford, Oxford, UK; NIHR Imperial Clinical Research Facility and NIHR Imperial Biomedical Research Centre, London, UK; Oxford Vaccine Group, Department of Paediatrics, University of Oxford, Oxford, UK; Oxford University Hospitals NHS Foundation Trust, Oxford, UK; Oxford Vaccine Group, Department of Paediatrics, University of Oxford, Oxford, UK; Peter Medawar Building for Pathogen Research, Nuffield Dept of Clinical Medicine, University of Oxford, Oxford, UK; NIHR Oxford Biomedical Research Centre, Oxford, UK; Peter Medawar Building for Pathogen Research, Nuffield Dept of Clinical Medicine, University of Oxford, Oxford, UK; The Jenner Institute, Nuffield Department of Medicine, University of Oxford, Oxford, UK; Department of Infection, Harrison Wing and NIHR Clinical Research Facility, Guys and St Thomas’ NHS Trust, London, UK; Department of Infection, Harrison Wing and NIHR Clinical Research Facility, Guys and St Thomas’ NHS Trust, London, UK; Department of Infection, Harrison Wing and NIHR Clinical Research Facility, Guys and St Thomas’ NHS Trust, London, UK; The Jenner Institute, Nuffield Department of Medicine, University of Oxford, Oxford, UK; Peter Medawar Building for Pathogen Research, Nuffield Dept of Clinical Medicine, University of Oxford, Oxford, UK; Peter Medawar Building for Pathogen Research, Nuffield Dept of Clinical Medicine, University of Oxford, Oxford, UK; Department of HIV Medicine, St Mary's Hospital, Imperial College Healthcare NHS Trust, London, UK; NIHR Oxford Biomedical Research Centre, Oxford, UK; Oxford University Hospitals NHS Foundation Trust, Oxford, UK; Peter Medawar Building for Pathogen Research, Nuffield Dept of Clinical Medicine, University of Oxford, Oxford, UK; Oxford University Hospitals NHS Foundation Trust, Oxford, UK; Centre for Tropical Medicine and Global Health, Nuffield Department of Medicine, University of Oxford, Oxford, UK; Mahidol-Oxford Tropical Medicine Research Unit, Mahidol University, Bangkok, Thailand; Peter Medawar Building for Pathogen Research, Nuffield Dept of Clinical Medicine, University of Oxford, Oxford, UK; Oxford University Hospitals NHS Foundation Trust, Oxford, UK; Department of Paediatrics, University of Oxford, Oxford, UK; Peter Medawar Building for Pathogen Research, Nuffield Dept of Clinical Medicine, University of Oxford, Oxford, UK; NIHR Oxford Biomedical Research Centre, Oxford, UK; Oxford University Hospitals NHS Foundation Trust, Oxford, UK; Department of Infectious Disease, Faculty of Medicine, Imperial College London, London, UK; Department of HIV Medicine, St Mary's Hospital, Imperial College Healthcare NHS Trust, London, UK; The Jenner Institute, Nuffield Department of Medicine, University of Oxford, Oxford, UK; The Jenner Institute, Nuffield Department of Medicine, University of Oxford, Oxford, UK; Wellcome Centre for Human Genetics, Nuffield Department of Medicine, University of Oxford, Oxford, UK; Public Health England, Porton Down, UK; Oxford Vaccine Group, Department of Paediatrics, University of Oxford, Oxford, UK; NIHR Oxford Biomedical Research Centre, Oxford, UK; Oxford Vaccine Group, Department of Paediatrics, University of Oxford, Oxford, UK; The Jenner Institute, Nuffield Department of Medicine, University of Oxford, Oxford, UK; Chinese Academy of Medical Sciences Oxford Institute, Oxford, UK; Peter Medawar Building for Pathogen Research, Nuffield Dept of Clinical Medicine, University of Oxford, Oxford, UK; Peter Medawar Building for Pathogen Research, Nuffield Dept of Clinical Medicine, University of Oxford, Oxford, UK; NIHR Oxford Biomedical Research Centre, Oxford, UK; Oxford University Hospitals NHS Foundation Trust, Oxford, UK

**Keywords:** SARS-CoV-2, COVID-19, Vaccination, Immune Response, HIV, People with HIV, T cell, Antibody

## Abstract

**Background:**

People with HIV on antiretroviral therapy with good CD4 T cell counts make effective immune responses following vaccination against SARS-CoV-2. There are few data on longer term responses and the impact of a booster dose.

**Methods:**

Adults with HIV were enrolled into a single arm open label study. Two doses of ChAdOx1 nCoV-19 were followed twelve months later by a third heterologous vaccine dose. Participants had undetectable viraemia on ART and CD4 counts >350 cells/µl. Immune responses to the ancestral strain and variants of concern were measured by anti-spike IgG ELISA, MesoScale Discovery (MSD) anti-spike platform, ACE-2 inhibition, Activation Induced Marker (AIM) assay and T cell proliferation.

**Findings:**

54 participants received two doses of ChAdOx1 nCoV-19. 43 received a third dose (42 with BNT162b2; 1 with mRNA-1273) one year after the first dose. After the third dose, total anti-SARS-CoV-2 spike IgG titres (MSD), ACE-2 inhibition and IgG ELISA results were significantly higher compared to Day 182 titres (P < 0.0001 for all three). SARS-CoV-2 specific CD4+ T cell responses measured by AIM against SARS-CoV-2 S1 and S2 peptide pools were significantly increased after a third vaccine compared to 6 months after a first dose, with significant increases in proliferative CD4 + and CD8+ T cell responses to SARS-CoV-2 S1 and S2 after boosting. Responses to Alpha, Beta, Gamma, and Delta variants were boosted, although to a lesser extent for Omicron.

**Conclusions:**

In PWH receiving a third vaccine dose, there were significant increases in B and T cell immunity, including to known VOCs.

## Teaser

People with Human Immunodeficiency Virus (HIV) who are on Antiretroviral Therapy (ART) with suppressed viraemia and CD4 counts >350 cells/ul, and who receive a third vaccine dose against SARS-CoV-2, make significant increases in antigen-specific B and T cell immune responses, including to known variants of concern (VOC).

## Supplementary Material

ciac796_Supplementary_Data

