# Booster Vaccination Against SARS-CoV-2 Induces Potent Immune Responses in People With Human Immunodeficiency Virus

**DOI:** 10.1093/cid/ciac796

**Published:** 2022-10-05

**Authors:** Sarah Fidler, Julie Fox, Timothy Tipoe, Stephanie Longet, Tom Tipton, Movin Abeywickrema, Sandra Adele, Jasmini Alagaratnam, Mohammad Ali, Parvinder K Aley, Suhail Aslam, Anbhu Balasubramanian, Anna Bara, Tanveer Bawa, Anthony Brown, Helen Brown, Federica Cappuccini, Sophie Davies, Jamie Fowler, Leila Godfrey, Anna L Goodman, Kathrine Hilario, Carl-Philipp Hackstein, Moncy Mathew, Yama F Mujadidi, Alice Packham, Claire Petersen, Emma Plested, Katrina M Pollock, Maheshi N Ramasamy, Hannah Robinson, Nicola Robinson, Patpong Rongkard, Helen Sanders, Teona Serafimova, Niamh Spence, Anele Waters, Danielle Woods, Panagiota Zacharopoulou, Eleanor Barnes, Susanna Dunachie, Philip Goulder, Paul Klenerman, Alan Winston, Adrian V S Hill, Sarah C Gilbert, Miles Carroll, Andrew J Pollard, Teresa Lambe, Ane Ogbe, John Frater

**Affiliations:** Department of Infectious Disease, Faculty of Medicine, Imperial College London, London, United Kingdom; Department of HIV Medicine, St Mary's Hospital, Imperial College Healthcare National Health Service (NHS) Trust, London, United Kingdom; National Institute for Health and Care Research (NIHR) Imperial Clinical Research Facility and NIHR Imperial Biomedical Research Centre, London, United Kingdom; NIHR Guy's and St Thomas’ Biomedical Research Centre, London, United Kingdom; Department of Infection, Harrison Wing and NIHR Clinical Research Facility, Guys and St Thomas’ NHS Trust, London, United Kingdom; Nuffield Department of Clinical Medicine, Peter Medawar Building for Pathogen Research, University of Oxford, Oxford, United Kingdom; Nuffield Department of Medicine, Wellcome Centre for Human Genetics, University of Oxford, Oxford, United Kingdom; Nuffield Department of Medicine, Wellcome Centre for Human Genetics, University of Oxford, Oxford, United Kingdom; Department of Infection, Harrison Wing and NIHR Clinical Research Facility, Guys and St Thomas’ NHS Trust, London, United Kingdom; Nuffield Department of Clinical Medicine, Peter Medawar Building for Pathogen Research, University of Oxford, Oxford, United Kingdom; Department of Infectious Disease, Faculty of Medicine, Imperial College London, London, United Kingdom; Department of HIV Medicine, St Mary's Hospital, Imperial College Healthcare National Health Service (NHS) Trust, London, United Kingdom; Nuffield Department of Clinical Medicine, Peter Medawar Building for Pathogen Research, University of Oxford, Oxford, United Kingdom; Oxford Vaccine Group, Department of Pediatrics, University of Oxford, Oxford, United Kingdom; Department of Infection, Harrison Wing and NIHR Clinical Research Facility, Guys and St Thomas’ NHS Trust, London, United Kingdom; Department of Infection, Harrison Wing and NIHR Clinical Research Facility, Guys and St Thomas’ NHS Trust, London, United Kingdom; National Institute for Health and Care Research (NIHR) Imperial Clinical Research Facility and NIHR Imperial Biomedical Research Centre, London, United Kingdom; Department of Infection, Harrison Wing and NIHR Clinical Research Facility, Guys and St Thomas’ NHS Trust, London, United Kingdom; Nuffield Department of Clinical Medicine, Peter Medawar Building for Pathogen Research, University of Oxford, Oxford, United Kingdom; Nuffield Department of Clinical Medicine, Peter Medawar Building for Pathogen Research, University of Oxford, Oxford, United Kingdom; Nuffield Department of Medicine, The Jenner Institute, University of Oxford, Oxford, United Kingdom; Nuffield Department of Medicine, The Jenner Institute, University of Oxford, Oxford, United Kingdom; Nuffield Department of Medicine, The Jenner Institute, University of Oxford, Oxford, United Kingdom; Nuffield Department of Medicine, The Jenner Institute, University of Oxford, Oxford, United Kingdom; Department of Infection, Harrison Wing and NIHR Clinical Research Facility, Guys and St Thomas’ NHS Trust, London, United Kingdom; Medical Research Council Clinical Trials Unit, University College London, London, United Kingdom; Department of Infection, Harrison Wing and NIHR Clinical Research Facility, Guys and St Thomas’ NHS Trust, London, United Kingdom; Nuffield Department of Clinical Medicine, Peter Medawar Building for Pathogen Research, University of Oxford, Oxford, United Kingdom; Department of Infection, Harrison Wing and NIHR Clinical Research Facility, Guys and St Thomas’ NHS Trust, London, United Kingdom; Nuffield Department of Medicine, The Jenner Institute, University of Oxford, Oxford, United Kingdom; Department of Infection, Harrison Wing and NIHR Clinical Research Facility, Guys and St Thomas’ NHS Trust, London, United Kingdom; Department of Infectious Disease, Faculty of Medicine, Imperial College London, London, United Kingdom; Department of HIV Medicine, St Mary's Hospital, Imperial College Healthcare National Health Service (NHS) Trust, London, United Kingdom; Nuffield Department of Medicine, The Jenner Institute, University of Oxford, Oxford, United Kingdom; National Institute for Health and Care Research (NIHR) Imperial Clinical Research Facility and NIHR Imperial Biomedical Research Centre, London, United Kingdom; Oxford Vaccine Group, Department of Pediatrics, University of Oxford, Oxford, United Kingdom; Oxford University Hospitals NHS Foundation Trust, Oxford, United Kingdom; Oxford Vaccine Group, Department of Pediatrics, University of Oxford, Oxford, United Kingdom; Nuffield Department of Clinical Medicine, Peter Medawar Building for Pathogen Research, University of Oxford, Oxford, United Kingdom; NIHR Oxford Biomedical Research Centre, Oxford, United Kingdom; Nuffield Department of Clinical Medicine, Peter Medawar Building for Pathogen Research, University of Oxford, Oxford, United Kingdom; Nuffield Department of Medicine, The Jenner Institute, University of Oxford, Oxford, United Kingdom; Department of Infection, Harrison Wing and NIHR Clinical Research Facility, Guys and St Thomas’ NHS Trust, London, United Kingdom; Department of Infection, Harrison Wing and NIHR Clinical Research Facility, Guys and St Thomas’ NHS Trust, London, United Kingdom; Department of Infection, Harrison Wing and NIHR Clinical Research Facility, Guys and St Thomas’ NHS Trust, London, United Kingdom; Nuffield Department of Medicine, The Jenner Institute, University of Oxford, Oxford, United Kingdom; Nuffield Department of Clinical Medicine, Peter Medawar Building for Pathogen Research, University of Oxford, Oxford, United Kingdom; Nuffield Department of Clinical Medicine, Peter Medawar Building for Pathogen Research, University of Oxford, Oxford, United Kingdom; Department of HIV Medicine, St Mary's Hospital, Imperial College Healthcare National Health Service (NHS) Trust, London, United Kingdom; Oxford University Hospitals NHS Foundation Trust, Oxford, United Kingdom; NIHR Oxford Biomedical Research Centre, Oxford, United Kingdom; Nuffield Department of Clinical Medicine, Peter Medawar Building for Pathogen Research, University of Oxford, Oxford, United Kingdom; Oxford University Hospitals NHS Foundation Trust, Oxford, United Kingdom; Nuffield Department of Medicine, Centre for Tropical Medicine and Global Health, University of Oxford, Oxford, United Kingdom; Mahidol-Oxford Tropical Medicine Research Unit, Mahidol University, Bangkok, Thailand; Nuffield Department of Clinical Medicine, Peter Medawar Building for Pathogen Research, University of Oxford, Oxford, United Kingdom; Oxford University Hospitals NHS Foundation Trust, Oxford, United Kingdom; Department of Paediatrics, University of Oxford, Oxford, United Kingdom; Nuffield Department of Clinical Medicine, Peter Medawar Building for Pathogen Research, University of Oxford, Oxford, United Kingdom; Oxford University Hospitals NHS Foundation Trust, Oxford, United Kingdom; NIHR Oxford Biomedical Research Centre, Oxford, United Kingdom; Department of Infectious Disease, Faculty of Medicine, Imperial College London, London, United Kingdom; Department of HIV Medicine, St Mary's Hospital, Imperial College Healthcare National Health Service (NHS) Trust, London, United Kingdom; Nuffield Department of Medicine, The Jenner Institute, University of Oxford, Oxford, United Kingdom; Nuffield Department of Medicine, The Jenner Institute, University of Oxford, Oxford, United Kingdom; Nuffield Department of Medicine, Wellcome Centre for Human Genetics, University of Oxford, Oxford, United Kingdom; Public Health England, Porton Down, Salisbury, United Kingdom; Nuffield Department of Medicine, The Jenner Institute, University of Oxford, Oxford, United Kingdom; NIHR Oxford Biomedical Research Centre, Oxford, United Kingdom; Oxford Vaccine Group, Department of Pediatrics, University of Oxford, Oxford, United Kingdom; Nuffield Department of Medicine, The Jenner Institute, University of Oxford, Oxford, United Kingdom; Chinese Academy of Medical Sciences Oxford Institute, Oxford, United Kingdom; Nuffield Department of Clinical Medicine, Peter Medawar Building for Pathogen Research, University of Oxford, Oxford, United Kingdom; Nuffield Department of Clinical Medicine, Peter Medawar Building for Pathogen Research, University of Oxford, Oxford, United Kingdom; Oxford University Hospitals NHS Foundation Trust, Oxford, United Kingdom; NIHR Oxford Biomedical Research Centre, Oxford, United Kingdom

**Keywords:** SARS-CoV-2, vaccination, people with HIV, T-cell responses, antibody responses

## Abstract

**Background:**

People with human immunodeficiency virus (HIV) on antiretroviral therapy (ART) with good CD4 T-cell counts make effective immune responses following vaccination against severe acute respiratory syndrome coronavirus 2 (SARS-CoV-2). There are few data on longer term responses and the impact of a booster dose.

**Methods:**

Adults with HIV were enrolled into a single arm open label study. Two doses of ChAdOx1 nCoV-19 were followed 12 months later by a third heterologous vaccine dose. Participants had undetectable viraemia on ART and CD4 counts >350 cells/µL. Immune responses to the ancestral strain and variants of concern were measured by anti-spike immunoglobulin G (IgG) enzyme-linked immunosorbent assay (ELISA), MesoScale Discovery (MSD) anti-spike platform, ACE-2 inhibition, activation induced marker (AIM) assay, and T-cell proliferation.

**Findings:**

In total, 54 participants received 2 doses of ChAdOx1 nCoV-19. 43 received a third dose (42 with BNT162b2; 1 with mRNA-1273) 1 year after the first dose. After the third dose, total anti-SARS-CoV-2 spike IgG titers (MSD), ACE-2 inhibition, and IgG ELISA results were significantly higher compared to Day 182 titers (*P* < .0001 for all 3). SARS-CoV-2 specific CD4+ T-cell responses measured by AIM against SARS-CoV-2 S1 and S2 peptide pools were significantly increased after a third vaccine compared to 6 months after a first dose, with significant increases in proliferative CD4+ and CD8+ T-cell responses to SARS-CoV-2 S1 and S2 after boosting. Responses to Alpha, Beta, Gamma, and Delta variants were boosted, although to a lesser extent for Omicron.

**Conclusions:**

In PWH receiving a third vaccine dose, there were significant increases in B- and T-cell immunity, including to known variants of concern (VOCs).

## Teaser

People with human immunodeficiency virus (HIV) on antiretroviral therapy (ART) with good CD4 T-cell counts make effective cross variant immune responses following third dose vaccination against severe acute respiratory syndrome coronavirus 2 (SARS-CoV-2).

Currently licensed vaccines targeting severe acute respiratory syndrome coronavirus 2 (SARS-CoV-2) protect against severe coronavirus disease 2019 (COVID-19) disease [1–6]. They induce robust humoral and cellular immunity against SARS-CoV-2 [2, 4, 7, 8], although with evidence of waning 6–8 months following vaccination [9–11]. The emergence of variants of concern (VOCs) including the B.1.1.7 (Alpha), B.1.351 (Beta), P.1 (Gamma), B.1.617.2 (Delta), and more recently the B.1.1.529 (Omicron) lineages showing increasing numbers of mutations [12, 13], high transmissibility [14, 15], immune escape [16–20], and increased incidence of breakthrough infections [21, 22] is particularly relevant to vulnerable populations [23], including people with human immunodeficiency virus (HIV, PWH). These factors contributed to the recommendation of a third dose of COVID-19 vaccine by some countries [10, 24–26].

For PWH, there is evidence for poorer immune responses and more severe clinical outcomes following infection with other non-related pathogens, including SARS-CoV-2 [27–33]. This can be partially rescued through antiretroviral therapy (ART)-mediated reconstitution of CD4 T-cell counts and T-cell effector function [34]. We recently demonstrated that similar to HIV seronegative individuals, PWH make potent T- and B-cell immune responses following 2 doses of ChAdOx1 nCoV-19 vaccination [3, 9], although with evidence of declining immunity at 6 months.

Third dose boosting with either homologous or heterologous combinations of COVID-19 vaccines results in vigorous immune responses [35, 36]. A third dose of BNT162b2 protected against infection and severe COVID-19 disease in adults >60 years of age [37]. For PWH, the increased immune responses afforded by booster vaccination may therefore offer protection, help overcome antigenic variation seen in some SARS-CoV-2 strains [38], and reduce the incidence of COVID-19.

We performed qualitative and quantitative assessment of humoral and cellular immune responses to SARS-CoV-2 and circulating VOCs following a third booster dose vaccine in PWH.

## METHODS

### Study Design and Cohort

The cohort has been described previously [3]. The study comprised people with HIV in an open-label non-randomized group within the larger multicentre phase 2/3 COV002 trial. Inclusion criteria were age 18–55 years, a diagnosis of HIV infection, virological suppression on ART at enrollment (plasma HIV viral load [VL] <50 copies per mL), and a CD4 count >350 cells/μL. Participants received 2 standard intramuscular doses of the ChAdOx1 nCoV-19 vaccine 4–6 weeks apart, and a third dose of any licensed COVID-19 vaccine after 1 year.

Participants with a history of laboratory-confirmed SARS-CoV-2 infection by anti-N protein immunoglobulin G (IgG) immunoassay (Abbott Architect, Abbott Park, Illinois, USA) at screening were excluded. Participants self-reported COVID-19 infection. Visits on day 0 (pre-ChAdOx1 nCoV-19 vaccine prime), 182 and “Post-Third Dose” were the main study timepoints for immunological analysis. As some participants did not attend their “Post-Third Dose” visit as they were lost to follow-up, there is a maximum of n = 43 at this timepoint. Where possible, we collected peripheral blood mononuclear cells (PBMCs) from participants before and after the third dose booster vaccine dose (n = 9).

### SARS CoV-2 Spike IgG ELISA

Humoral responses at baseline and following vaccination were assessed using a standardized total IgG enzyme-linked immunosorbent assay (ELISA) against SARS CoV-2 spike as described previously [2]. Full details are in Supplementary Materials.

### Mesoscale Discovery (MSD) Binding Assays

IgG responses to SARS-CoV-2 variant spike antigens including Wuhan strain, Alpha, Beta, Gamma, Delta, and Omicron were measured using a multiplexed V-PLEX COVID-19 Coronavirus Panel 23 Kit (K15570U-2) from Meso Scale Diagnostics, Rockville, Maryland, USA. Full details are in Supplementary Materials.

### T-Cell Proliferation Assay

T-cell proliferation was measured use a CTV assay [3, 9]. Full details are in Supplementary Table 2.

### AIM Assay

The activation induced marker (AIM) assay was used to identify and characterise antigen-specific T cells [3, 9]. Full details are in Supplementary Table 3.

### ACE-2 Inhibition Assay

A multiplexed MSD immunoassay (MSD, Rockville, Maryland, USA) was used to measure the ability of human sera to inhibit ACE-2 binding to SARS-CoV-2 spike (B, B.1, B.1.1.7, B.1.351 or P.1, B.1.617, B.1.1.59). Full details are in Supplementary Materials.

### Statistical Analysis

We analyzed all outcomes in all participants who received specified doses of the vaccination schedule and with available samples, unless otherwise specified. We present medians and interquartile ranges (IQRs) for immunological endpoints. For comparison of 2 non-parametrically distributed unpaired variables, we used the Wilcoxon rank sum (Mann-Whitney *U*) test. Where multiple data points were compared, we used a Kruskal-Wallis test with Dunn's multiple comparison. For comparison of 2 non-parametrically distributed paired data sets, we used the Wilcoxon matched pairs signed rank test. All analyses were carried out using Prism 9 (GraphPad Software).

### Study Approval

Study approval in the United Kingdom was by the Medicines and Healthcare products Regulatory Agency (reference 21584/0424/001-0001) and the South Central Berkshire Research Ethics Committee (reference 20/SC/0145). COV002 is registered with ClinicalTrials.gov, NCT04400838.

## RESULTS

### Participants

**Figure 1.**
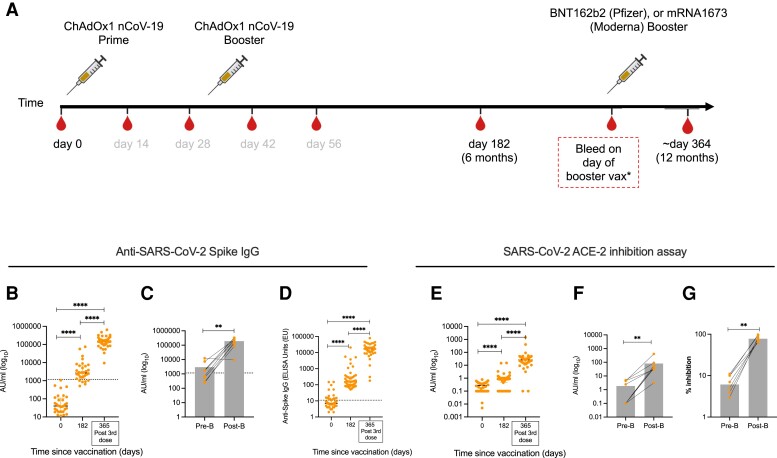
Anti-SARS-CoV-2 antibody responses are boosted following third dose of COVID-19 vaccines in PWH. *A*, Vaccination schedule for all participants showing timepoints where samples were used for this study in black. PWH received either BNT162b2, mRNA1273, or ChAdOx1 nCoV-19 vaccines. The third dose was given as close to 1 y after the first vaccine dose as possible. The “Day 365” visit sample was the “post-third dose” sample”. *B*, Anti-SARS-CoV-2 spike IgG antibody titers before priming vaccine dose at day 0 and post-prime doses at day 182 and 365 (after third dose). *C*, Anti-SARS-CoV-2 spike IgG antibody titers in HIV positive participants with pre- and post-third dose timepoints. *D*, In-house ELISA showing anti-spike IgG responses at baseline, day 182 and after third dose (*E*) ACE-2 inhibition assay on day 0, 182, and after third dose in all participants. *F* and *G*, ACE-2 inhibition assay in participants with pre- and post-third dose timepoints expressed as *F*, AU/mL or *G*, % inhibition. Comparison of 2 timepoints within the same group was done by Wilcoxon matched pair sign ranked test. Where indicated * = *P* <.05, ** = *P* <.01, *** = *P* < .001 and **** = <.000. “Pre-B” and “Post-B” refer to pre-third dose and post-third dose. Dotted lines indicate cutoff points determined for each SARS-CoV-2 spike antigen based on pre-pandemic sera + 3 × SD. N = 27–33 for HIV positive volunteers in MSD assay. Error bars represent median and interquartile range. Abbreviations: COVID-19, coronavirus disease 2019; ELISA, enzyme-linked immunosorbent assay; HIV, human immunodeficiency virus; IgG, immunoglobulin G; MSD, MesoScale Discovery; PWH, people with HIV; SARS-CoV-2, severe acute respiratory syndrome coronavirus 2.

Participants with HIV (n = 54; all male) were recruited as a sub-study group in the COV002 clinical trial (NCT04400838) in November 2020. Participants were administered 2 doses of ChAdOx1 nCoV-19 vaccine at day 0 and after 4–6 weeks. They were offered a third dose with a heterologous vaccine around 365 days after their first ChAdOx1 nCoV-19 dose. All participants had undetectable VL (<50 HIV RNA copies/mL) and a median CD4 count of 694 cells/µL (IQR: 573.5–859.5) at the time of recruitment. Ethnicity was mostly White (81.5%). Other reported ethnicities were Asian (3.7%), mixed (7.4%), and other (7.4%). Participants returned for study visits on day 14, 28, 42, 56, 182, and “Post-Third Dose,” The “Post-Third Dose” visit was recorded as the first study visit following the third dose of vaccine (mean number of days post third dose = 33, range: 5–115, IQR: 21–41). Participants received mostly BNT162b2 vaccine for their day 365 boost (42/43; 1/43 received mRNA-1273; Moderna) (Supplementary Table 1). For this study, baseline (Day 0), 6 months (Day 182), and “Post-Third Dose” samples were considered. The introduction of the booster vaccine as National Health Service (NHS) policy by the UK government meant some third doses were given out of sync with the study protocol, and so blood draws before the third dose were not available for all participants. However, for some (n = 9), samples were available either side of the third dose, as pre- and post-third dose visits (Figure 1*A* and Table 1). All participants self–reported an absence of SARS-CoV-2 infection at every study visit based on interviews with the study team, and SARS-CoV-2 nucleocapsid responses measured for 6 months after recruitment.

**Table 1.** Demographic Information for Participants Used in This Study

Participant Summary		
Sex	Male	54 (100%)
	Female	0 (0%)
Age in years		42.5 (37.2–49.8)
Ethnicity	White	44 (81.5%)
	Black	0 (0%)
	Asian	2 (3.7%)
…	Mixed	4 (7.4%)
…	Other	4 (7.4%)
Antiretroviral therapy	Y	54 (100%)
	N	…
Plasma HIV VL (copies/mL)		<50
CD4 count > 350 cells/uL		694.0 (573.5–859.5)^b^
Nadir CD4 count (cells/uL)^a^		366 (220–514)^b^

Abbreviations: HIV, human immunodeficiency virus; IQR, interquartile range; VL, viral load.

Data available for n = 31.

Median (IQR).

### Antibody Responses to SARS-CoV-2 Are Boosted Following a Third Dose of COVID-19 Vaccine in PWH

The MesoScale Discovery (MSD) assay platform was used to quantify plasma levels of circulating total anti-SARS-CoV-2 spike IgG. We previously reported that anti-spike IgG and pseudo-neutralizing antibody levels 182 days after first vaccination were significantly higher than baseline levels measured on day 0 [13]. Analysis of plasma samples “Post-Third Dose” showed that total anti-SARS-CoV-2 spike IgG titers were significantly higher than day 182 titers (n = 32; day 182 = median 2644 (IQR: 1341–6614) AU/mL, post-third dose = median 143 088 (IQR 96 854–189 674) AU/mL; *P* < .0001), and to an even higher degree when compared to baseline levels (n = 32; day 0 = median 40 (IQR: 19.5–109.6) AU/mL; *P* < .0001) (Figure 1*B*). To further evaluate the impact of a third dose on antibody levels, we measured anti-SARS-CoV-2 spike IgG in plasma of the subset of 9 participants with both pre- and post-third boost samples. We found a significant increase in anti-SARS-CoV-2 spike IgG titers in participants following booster vaccination (pre-boost = median 1714 (IQR: 417–4622) AU/mL; post-boost = median 188 590 (IQR: 104 806–290 778) AU/mL; *P* = .0078) (Figure 1*C*, and Supplementary Figure 1*A*). Antibodies against the SARS-CoV-2 spike protein were also measured by IgG ELISA, which supported the increased response after a third dose. IgG responses peaked 42 days after the first of the 2 initial vaccine doses (median 1440 ELISA units [EU], IQR: 704–2728; n = 50) but had reduced significantly by the 6 months timepoint (median 158 ELISA units [EU], IQR: 88–325; n = 47) (*P* < .0001) (Supplementary Figure 1*B*). After the third vaccine dose the response was significantly boosted (median 17 025 ELISA units [EU], IQR: 10 634–22 847; n = 43)(*P* < .0001) (Figure 1*D*).

Next, we evaluated the level of antibodies capable of out-competing the binding of SARS-CoV-2 to human ACE-2 to prevent viral entry in an ACE-2 inhibition assay, a surrogate of antibody neutralisation. We found significantly higher titers of antibodies capable of blocking ACE-2 binding of SARS-CoV-2 “Post-Third Dose” visit compared to day 182 and day 0 (n = 27, day 0 = median 0.39 (IQR: 0.253–0.50) AU/mL, day 182 median 0.99 (IQR: 0.83–1.37) AU/mL, “post-third dose” median 27.15 (IQR: 15.36–42.77) AU/mL) (Figure 1*E*). This booster effect of the vaccination was confirmed in participants with pre- and post-boost timepoints (n = 9 pre-boost median 0.1 (IQR: 0.1–4.44) AU/mL, post-boost median 37.05 (IQR: 30.42–73.1) AU/mL) (Figure 1*F,*1*G*, and Supplementary Figure 1*C*). We did not observe any correlations between the number of days post-boost and antibody titers or ACE-2 binding inhibition (data not shown).

### Increased Magnitude of T-Cell Responses After Third COVID-19 Vaccine Dose in PWH

T-cell immune responses were first measured using an ex vivo AIM assay to measure effector-type responses and then a CTV proliferation assay on 7-day expanded cells to quantify recall response. (Flow cytometric gating strategy for AIM and proliferation assays are shown in Supplementary Figures 2*A* and *F*, respectively**)**. Staphylococcal enterotoxin B (SEB) and cytomegalovirus (CMV) responses were used as mitogenic and antigenic control responses in the AIM assay (Supplementary Figure 2*B*–*E*), whereas phytohemagglutinin (PHA) and Flu, EBV, CMV, Tetanus (FECT) optimal peptides were used as controls in the proliferation assays (Supplementary Figure 2*G*–*J*).

**Figure 2.**
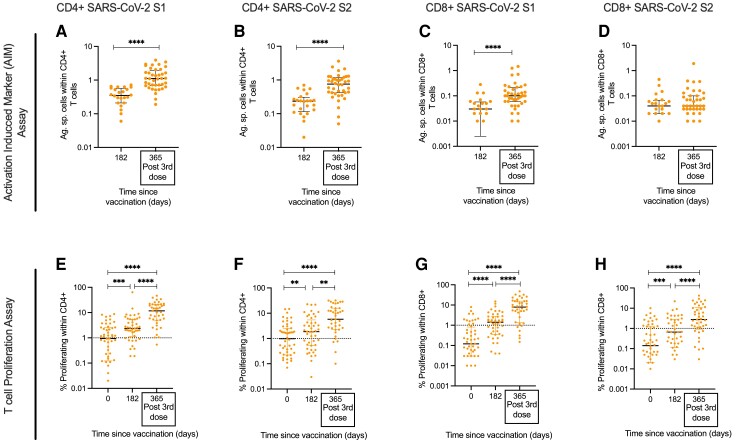
T-cell response to SARS-CoV-2 following third dose of COVID-19 vaccines in PWH. T-cell responses measured by AIM assay showing magnitude of CD4+ T-cell responses to (*A*) SARS-CoV-2 S1 and (*B*) SARS-CoV-2 S2 and magnitude of CD8+ T-cell responses to (*C*) SARS-CoV-2 S1 and (*D*) SARS-CoV-2 S2 on days 182 and after third dose (D365). Proliferative T-cell responses assessing kinetics of the T-cell response longitudinally for CD4+ T cells to (*E*) SARS-CoV-2 S1 and (*F*) SARS-CoV-2 S2 and CD8+ T cells to (*G*) SARS-CoV-2 S1 and (*H*) SARS-CoV-2 S2. Statistical test in (*A*–*D*) was done by Mann-Whitney *t* test. Statistical test in (*E*–*H*) was done by Wilcoxon matched pair sign ranked test. Where indicated * = *P* <.05, ** = *P* <.01, *** = *P* < .001 and **** = *P* <.000. Dotted lines indicate cutoff points determined based on DMSO controls + 3 × SD. n = 24–40 for AIM assay and 41–52 for proliferation assay. Error bars represent median and interquartile range. Abbreviations: AIM, activation induced marker; DMSO, dimethyl sulfoxide; PWH, people with HIV; SARS-CoV-2, severe acute respiratory syndrome coronavirus 2; SD, standard deviation.

The AIM assay showed that the frequency of SARS-CoV-2 specific CD4+ T-cell responses against SARS-CoV-2 S1 and S2 peptide pools was significantly increased by >3-fold after a third vaccine compared to their levels 6 months post ChAdOx1 nCoV-19 prime (CD4+ SARS-CoV-2 S1: day 182 median 0.35% (IQR: 0.21–0.56), post-third dose median 1.11% (IQR: 0.68–3.93); CD4+ SARS-CoV-2 S2: day 182 median 0.235% (IQR: 0.12–0.3), post-third dose median 0.76% (IQR 0.42–1.17)) (Figure 2*A* and 2*B*). The frequency of AIM+ SARS-CoV-2 specific CD8+ T cells targeting SARS-CoV-2 S1 but not S2 significantly increased at the “Post-Third Dose” visit compared to 6 months (CD8+ SARS-CoV-2 S1: day 182 = median 0.03% (IQR: 0.003–0.057), post-third dose median 0.1% (IQR: 0.06–0.21); CD8+ SARS-CoV-2 S2: day 182 median 0.04% (IQR: 0.02–0.066), post-third dose median 0.04% (IQR: 0.03–0.1) (Figure 2*C* and 2*D*).

These observed T-cell responses from the AIM assay were also seen when measuring T-cell proliferation, although with a greater magnitude. Proliferative CD4+ and CD8+ T-cell responses to SARS-CoV-2 S1 and S2 following the third dose were significantly greater than responses at baseline (day 0) and day 182 after first dose (Figure 2*E*–*H*). Analysis of the magnitude of the CD4+ and CD8+ proliferative response following vaccination showed that T-cell responses were primed after initial vaccine, peaking between days 28 and 42, had waned by day 182 [13], and then increased again following the third dose (Supplementary Figure 3*A*–*H*). These assays indicate potent boosting of T-cell responses by vaccination and efficient recall upon antigen re-exposure.

### Phenotypic Analysis of SARS-CoV-2 Specific Cells Following Booster Vaccination

**Figure 3.**
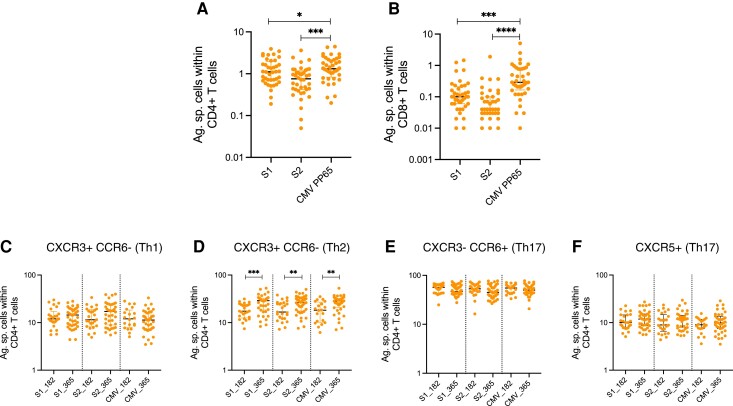
Phenotype of AIM+ antigen specific responses following third COVID-19 vaccine dose in PWH. Comparative analysis of the magnitude of antigen-specific T cells to SARS-CoV-2 S1, SARS-CoV-2 S2, and CMVpp65 in (*A*) CD4+ and (*B*) CD8+ T cells. Phenotype of antigen specific T cells 6 m after the priming ChAdOx1 nCoV-19 dose and after third heterologous dose showing (*C*) CXCR3+ CCR6-Th1, (*D*) CXCR3− CCR6-Th2, (*E*) CXCR3− CCR6+ Th17, and (*F*) CXCR5+ circulating Tfh CD4+ T cells. Statistical tests for (*A*) and (*B*) were done by Kruskal-Wallis with Dunn's multiple comparison. Statistical tests in (*C*–*F*) were done by Mann-Whitney *t* test. Where indicated * = *P* <.05, ** = *P* <.01, *** = *P* < .001, and **** = <.000. *n* = 20–40. Error bars represent median and interquartile range. Abbreviations: AIM, activation induced marker; COVID-19, coronavirus disease 2019; PWH, people with HIV; SARS-CoV-2, severe acute respiratory syndrome coronavirus 2.

As we had observed an increase in the magnitude of SARS-CoV-2 T cells following third dose vaccination, we assessed if there were changes in the distribution of the phenotype of the CD4+ T helper cell subsets following the booster vaccine. We first compared the magnitude of all antigen-specific cells within CD4 and CD8+ T-cell compartments using the AIM assay. We observed that despite the recent boost of SARS-CoV-2 spike-specific T cells, CMVpp65-specific T-cell response remained at a higher frequency compared to SARS-CoV-2 spike-specific responses (Figure 3*A* and 3*B*). We then used chemokine receptors CXCR3 and CCR6 to evaluate the distribution of CD4+ T-cell subsets within the antigen-specific AIM+ CD4+ T cells 6 months after priming vaccination and after the third dose. We found no change in the frequency of SARS-CoV-2 spike-specific CD4+ T cells that exhibited a Th1 (CXCR3+ CCR6−), Th17 (CXCR3− CCR6+) or circulating Tfh (CXCR5+) phenotype following a third dose (Figure 3*C*, *E*, and *F*). We noted an increase in the frequency of Th2 (CXCR3− CCR6−) cells within the CD4+ antigen-specific compartment; however, this was found with all antigens (including CMV) and, in the absence of functional data, larger studies would be needed to determine if this was reproducible (Figure 3*D*).

### Potent VOC Immune Responses Are Induced Following Booster Vaccines

**Figure 4.**
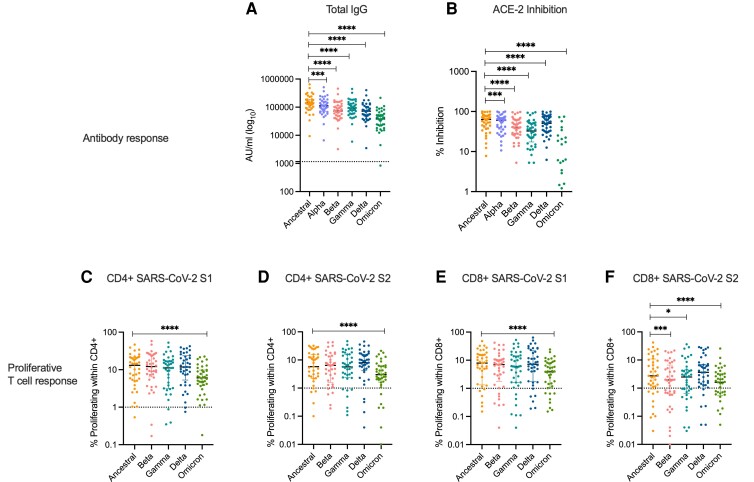
Immune response to SARS-CoV-2 VOC following third dose of COVID-19 vaccines in PWH. *A*, Total anti-spike IgG antibody responses (*B*) ACE-2 inhibition assay to circulating VOC. Proliferation assay comparing magnitudes of proliferative T-cell response to SARS-CoV-2 parental strain to a panel of VOC for CD4+ (*C*) SARS-CoV-2 S1, (*D*) SARS-CoV-2 S2 and CD8+, (*E*) SARS-CoV-2 S1, (*F*) SARS-CoV-2 S2. Statistical tests were done by Wilcoxon matched pair sign ranked test. Where indicated * = *P* <.05, ** = *P* <.01, *** = *P* < .001 and **** = *P* <.000. Dotted lines indicate cutoff points determined for each SARS-CoV-2 spike antigen based on pre-pandemic sera + 3 × SD for antibody responses and cutoff points determined based on DMSO controls + 3 × SD for proliferative responses. *n* = 37–40 for antibody analysis and n = 41 or proliferation assay. Error bars represent median and interquartile range. Abbreviations: DMSO, dimethyl sulfoxide; IgG, immunoglobulin G; PWH, people with HIV; SARS-CoV-2, severe acute respiratory syndrome coronavirus 2; SD, standard deviation; VOC, variants of concern.

Finally, we evaluated the magnitude of humoral and T-cell responses to circulating VOCs (including the recently categorized Omicron BA1 variant) after a third dose. Compared to total anti-SARS-CoV-2 spike IgG titers in the ancestral strain, total anti-spike antibody responses to all VOCs were significantly reduced (Figure 4*A*). This was also found with the SARS-CoV-2 ACE-2 binding assay, which indicated a decreased potency of neutralising antibodies in the “Post-Third Dose” sample to bind to spike protein from VOCs (Figure 4*B*). For VOCs—Alpha, Beta, and Gamma—for which we had historical day 0 and day 182 data, we assessed the kinetics of the antibody response after the third dose. We noted a striking increase in ACE inhibition (Supplementary Figure 1*D*–*F*) and antibody titers (Supplementary Figure 4*A*–*C*). after the third dose compared to samples tested at baseline and 6 months after the first of the 2 ChAdOx1 nCoV-19 doses.

We also investigated T-cell responses to VOCs in comparison to the ancestral SARS-CoV-2 Victoria strain. Similar to our previous report [13], the magnitude of the proliferative CD4+ and CD8+ T-cell response was comparable between the ancestral strain and the Beta, Gamma, and Delta variants—with the exception of the CD8+ T-cell response to SARS-CoV-2 S2 peptide pool. Interestingly, we found the proliferative T-cell response to the Omicron variant targeting both spike S1 and S2 peptide pools was significantly reduced in the CD4 and CD8+ T-cell compartments (Figure 4*C*–*F*). Where sample availability allowed, we compared the kinetics of the T-cell response 6 months after first vaccination and after a third dose, and found an increase in T-cell responses to all variants tested after a third dose, with the sole exception of the CD8 T-cell response to the SARS-CoV-2 Beta variant S1 subunit (Supplementary Figure 4*D*–*O*).

As Omicron-directed antibody and T-cell responses were significantly lower than the responses to the ancestral SARS-CoV-2 strain, we looked in more detail in participants sampled before and shortly after their third dose of COVID-19 vaccine (n = 9). We found moderate but statistically significant increases in both humoral (Supplementary Figure 5*A* and *B*) and CD4+ and CD8+ T cell (Supplementary Figure 5*C* and *D*) responses to the Omicron variant after the third dose. Taken together our data show that booster vaccination in PWH significantly boosts antibody and T-cell responses to Alpha, Beta, Gamma, and Delta VOCs, and to a lesser extent to Omicron.

## DISCUSSION

We show evidence that a third dose of the licensed COVID-19 vaccines significantly boosted antibody and T-cell responses in PWH (VL undetectable and CD4 count >350 cells/µL). The robust responses generated in our cohort of PWH following heterologous third dose regimen are consistent with reports in people without HIV [39–41] and are reassuring, especially as the ChAdOx1 nCov-19 vaccine is well designed for distribution in low- to middle-income countries including those with a significant prevalence of PWH [42].

Equally crucial in the strategic management of the COVID-19 pandemic is that boosted SARS-CoV-2 immune responses can target circulating VOCs, especially as immune escape has been reported [16–18, 43]. We found humoral responses to VOCs to be boosted although to a lesser degree than responses targeting the ancestral strain. There was no difference between the magnitude of T-cell responses to the VOCs except for the Omicron variant, which was boosted but to lower levels than other VOCs. The relatively high number of mutations on key sites of antibody target including K417N and N501Y in the Omicron spike protein may account for this [13, 44]. Interestingly, our data may suggest that antibody immune evasion is more prevalent than T cell escape in immune response to VOCs—whether T cells may therefore play a role in protection from VOC-mediated COVID-19 needs further investigation [45]. Real world data would also be needed to determine if boosted VOC responses confer protection from severe COVID-19 disease in PWH. Finally, the quality of the induced immune response may be impacted by the vaccine platform. For example, there is evidence that the ChAdOx1 nCoV-19 vaccine results in a more dominant Th1-driven response [46] and mRNA vaccines may induce stronger antibody responses [47], possibly by soliciting Tfh cell help [48–50].

Our study has some limitations. We do not have access to a control group of HIV seronegative volunteers tested with the same assays in the same conditions post-boost, and so cannot comment on how the magnitude of immune response in our cohort of PWH would compare to HIV negative controls. We assessed breakthrough infection with SARS-CoV-2 by direct questioning of participants at every study visit. This was supported by nucleocapsid responses, but only for the first six months of the study. Our cohort of PWH represent the scenario of ART suppressed volunteers with an undetectable VL and high CD4 count. This is not the case for many PWH. As such, the data from our cohort should be extrapolated cautiously to other populations with HIV, especially as our cohort was also biaised to male participants in the United Kingdom. Due to the roll-out of the UK vaccination program during the study, we were only able to obtain pre-third dose samples from nine participants. It is therefore difficult to state exactly what the immediate increase in immune response was, although it is clear that the overall response was significantly augmented. Finally, as most participants received the BNT162b2 vaccine as the third dose after the two ChAdOx1 nCoV-19 doses, we did not have the scope to perform a comparative analysis of immune responses following a different third dose vaccine, which may be especially relevant in countries without access to RNA vaccines. In summary, we show a robust booster effect on antibody and T-cell responses to SARS-CoV-2 in PWH after a third dose in a heterologous vaccination schedule.

## Supplementary Data

Supplementary materials are available at *Clinical Infectious Diseases* online. Consisting of data provided by the authors to benefit the reader, the posted materials are not copyedited and are the sole responsibility of the authors, so questions or comments should be addressed to the corresponding author.

## Supplementary Material

ciac796_Supplementary_Data

## Notes

***Financial support*.** UK Research and Innovation, Engineering and Physical Sciences Research Council, National Institutes for Health Research (NIHR), Coalition for Epidemic Preparedness Innovations (CEPI), NIHR Oxford Biomedical Research Centre, Thames Valley and South Midlands NIHR Clinical Research Network, and AstraZeneca. The views expressed are those of the authors and not necessarily those of the NIHR or the Department of Health and Social Care. A. V. S. H. reports grants to Oxford University from AstraZeneca and Serum Institute of India in support of this work. A. J. P. reports support in the form of vaccines provided by Novavax (no financial support). K. M. P. reports support in the form of payments to institution from Medical Research Council/United Kingdom Research & Innovation (MRC/UKRI), the Vaccine Task Force, and NIHR Imperial BRC. P. K. A. and T. L. report grant to support the running of the trial paid to University of Oxford from Vaccine Taskforce via NIHR. C. P. H. reports WT109965MA, awarded to P. K. from Wellcome Trust, and Research Fellowship 403193363 awarded to C.-P. H. from Deutsche Forschungsgemeinschaft (DFG). S. L. reports US Food and Drug Administration Medical Countermeasures Initiative contract 75F40120C00085 to Miles Carroll's group. S. Du. reports being funded by an NIHR Global Research Professorship (NIHR300791), National Institute for Health & Care Research, United Kingdom.

***Potential conflicts of interest*.** A. V. S. H. reports being a potential beneficiary of royalties paid by AstraZeneca to Oxford University and a beneficiary of income from licenses to Serum Institute of India by Oxford University; is a named inventor on patent filed on COVID ChAdOx1 vaccine studied here; and is a stock owner in Vaccitech plc which is a beneficiary of royalties from the ChAdOx1 COVID vaccine. A. J. P. reports a role as Chair of UK Department of Health and Social Care's (DHSC) Joint Committee on Vaccination & Immunisation (JCVI), but does not participate in discussions on COVID-19 vaccines, and is a member of the World Health Organization’s (WHO) SAGE. The views expressed in this article do not necessarily represent the views of DHSC, JCVI, NIHR or WHO, a role as NIHR Senior Investigator for NIHR, and Oxford University has entered into a partnership with AstraZeneca for the development of a coronavirus vaccine. A. L. G. reports that her institutions (Guy's and St Thomas’ NHS Foundation Trust) receive grants and funding to deliver a range of COVID trials; honoraria have been unpaid or donated to charity immediately; is named as an inventor on a patent covering use of a particular promoter construct that is often used in ChAdOx1-vectored vaccines and is incorporated in the ChAdOx1 nCoV-19 vaccine and may benefit from royalty income paid to the University of Oxford from sales of this vaccine by AstraZeneca and its sublicensees under the University's revenue sharing policy. A. O. reports consulting fees from Genome BC, Canada and TakeTwo Interactive; payment or honoraria for lectures, presentations, speakers’ bureaus, manuscript writing or educational events from Babraham Institute, University of Cambridge; support for attending meetings and/or travel from Institute of Arts and Ideas and British Society for Immunology; stock or stock options from Adaptimmune. K. M. P. reports grants or contracts paid to institution from Chan Zuckerberg Initiative; consulting fees and participation as DSMB member for NCT05249829 from PPD for Moderna; payment as lecturer or for board of educational meeting from Seqirus, Sanofi Pasteur; support to attend meeting in Poland 2021 from Imperial College London; unpaid role as member of COVID-19 vaccine research chief investigator's group; and other financial or non-financial interests, paid to institution, as chief, principal or co-investigator for vaccine clinical trials and experimental medicine studies (NCT05007275, NCT04753892, EudraCT 2020-001646-20, NCT04400838, NCT04324606, EudraCT 2017-004610-26, NCT03970993, NCT03816137). M. N. R. reports interests as a PI on clinical trials sponsored and or funded by AstraZeneca but does not receive any personal financial payment for this work. P. K. A. reports grants unrelated to this work to support the running of the trial paid to University of Oxford from AstraZeneca and NIHR; and receipt of IMP from AstraZeneca. S. F. reports grants or contracts unrelated to this work from Imperial College NIHR BRC and NIHR ChAdOx; consulting fees from Gilead scientific advisory board paid to Imperial College London and from ImmunoCore Chief investigator paid to Imperial College NHS Trust; payment or honoraria for lectures, presentations, speakers’ bureaus, manuscript writing, or educational events from University of Ghent to Imperial College London; from University of Aarhus (personal) and from Gilead to Imperial College London; participation on HIV vaccine trial DSMB. S. C. G. reports COVID-19 vaccine development grants from UKRI, NIHR, CEPI to University of Oxford; expected future royalties or license payments from AstraZeneca for the COVID-19 vaccine, to be distributed by University of Oxford; patent on ChAdOx1, and on ChAdOx1 nCoV-19, held by University of Oxford and licensed to AstraZeneca; and is a co-founder of Vaccitech and holds stock (former consultant and board member). S. L. reports US Food and Drug Administration Medical Countermeasures Initiative contract 75F40120C00085 to M. C.’s group. S. Du. reports PITCH Consortium has received funding from UK Department of Health and Social Care as part of the PITCH (Protective Immunity from T cells to COVID-19 in Health workers) Consortium, UKRI as part of “Investigation of proven vaccine breakthrough by SARS-CoV-2 variants in established UK healthcare worker cohorts: SIREN consortium & PITCH Plus Pathway” MR/W02067X/1, with contributions from UKRI/NIHR through the UK Coronavirus Immunology Consortium (UK-CIC), the Huo Family Foundation, and the National Institute for Health & Care Research (UKRIDHSC COVID-19 Rapid Response Rolling Call, Grant Reference Number COV19-RECPLAS); grants or contracts from UKRI, Huo Family Foundation, and National Institute for Health & Care Research, United Kingdom; consulting fees as a Scientific Advisor to the Scottish Parliament on COVID-19; participation as a member of Wellcome's Clinical Interview Committee, 2021, from Wellcome Trust; a role as a member of the Treatment Guidelines Writing Group for Ebola with the WHO. T. L. reports consulting fees as a consultant to Vaccitech for an unrelated project; payment or honoraria for meeting relating to influenza meeting—unrelated work—from Seqirus; is named as an inventor on a patent application for a vaccine against SARS CoV-2; and work-related pension shares and ISAs. P. K. reports a grant from Pfizer Global for work on IBD; and consulting fees for participation on Advisory board for AstraZeneca on inflammation. E. B. reports patents in hepatitis B virus (HBV) and hepatitis C virus (HCV) vaccines in ChAdOx1; and participation as member of vaccitech scientific advisory board developing ChAOX1 vaccines in HBV. All other authors report no potential conflicts.

## References

[1] Ramasamy MN , Minassian AM, Ewer KJ, et al Safety and immunogenicity of ChAdOx1 nCoV-19 vaccine administered in a prime-boost regimen in young and old adults (COV002): a single-blind, randomised, controlled, phase 2/3 trial. Lancet 2020; 396:1979–93. 33220855 10.1016/S0140-6736(20)32466-1 PMC7674972

[2] Folegatti PM , Ewer KJ, Aley PK, et al Safety and immunogenicity of the ChAdOx1 nCoV-19 vaccine against SARS-CoV-2: a preliminary report of a phase 1/2, single-blind, randomised controlled trial. Lancet 2020; 396:467–78. 32702298 10.1016/S0140-6736(20)31604-4 PMC7445431

[3] Frater J , Ewer KJ, Ogbe A, et al Safety and immunogenicity of the ChAdOx1 nCoV-19 (AZD1222) vaccine against SARS-CoV-2 in HIV infection: a single-arm substudy of a phase 2/3 clinical trial. Lancet HIV 2021; 8:e474–85. 34153264 10.1016/S2352-3018(21)00103-X PMC8213361

[4] Walsh EE , Frenck RW, Falsey AR, et al Safety and immunogenicity of two RNA-based COVID-19 vaccine candidates. N Engl J Med 2020; 383:2439–50. 33053279 10.1056/NEJMoa2027906 PMC7583697

[5] Polack FP , Thomas SJ, Kitchin N, et al Safety and efficacy of the BNT162b2 mRNA COVID-19 vaccine. N Engl J Med 2020; 383:2603–15. 33301246 10.1056/NEJMoa2034577 PMC7745181

[6] Baden LR , El Sahly HM, Essink B, et al Efficacy and safety of the mRNA-1273 SARS-CoV-2 vaccine. N Engl J Med 2020; 384:403–16. 33378609 10.1056/NEJMoa2035389 PMC7787219

[7] Li J , Hui A, Zhang X, et al Safety and immunogenicity of the SARS-CoV-2 BNT162b1 mRNA vaccine in younger and older Chinese adults: a randomized, placebo-controlled, double-blind phase 1 study. Nat Med 2021; 27:1062–70. 33888900 10.1038/s41591-021-01330-9

[8] Voysey M , Costa Clemens SA, Madhi SA, et al Single-dose administration and the influence of the timing of the booster dose on immunogenicity and efficacy of ChAdOx1 nCoV-19 (AZD1222) vaccine: a pooled analysis of four randomised trials. Lancet 2021; 397:881–91. 33617777 10.1016/S0140-6736(21)00432-3 PMC7894131

[9] Ogbe A , Pace M, Bittaye M, et al Durability of ChAdOx1 nCov-19 vaccination in people living with HIV. JCI Insight 2022; 7:e157031. 35192543 10.1172/jci.insight.157031 PMC9057612

[10] Pegu A , O’Connell S, Schmidt SD, et al Durability of mRNA-1273 vaccine–induced antibodies against SARS-CoV-2 variants. Science 2021; 373:1372–7. 34385356 10.1126/science.abj4176 PMC8691522

[11] Widge AT , Rouphael NG, Jackson LA, et al Durability of responses after SARS-CoV-2 mRNA-1273 vaccination. N Engl J Med 2021; 384:80–2. 33270381 10.1056/NEJMc2032195 PMC7727324

[12] ECDC . Implications of the further emergence and spread of the SARS-CoV-2 B.1.1.529 variant of concern (Omicron) for the EU/EEA—first update. In: European Centre for Disease Prevention and Control. Brief Report.

[13] Karim SSA , Karim QA. Omicron SARS-CoV-2 variant: a new chapter in the COVID-19 pandemic. Lancet 2021; 398:2126–8. 34871545 10.1016/S0140-6736(21)02758-6 PMC8640673

[14] Volz E , Hill V, McCrone JT, et al Evaluating the effects of SARS-CoV-2 spike mutation D614G on transmissibility and pathogenicity. Cell 2021; 184:64–75.e11. 33275900 10.1016/j.cell.2020.11.020 PMC7674007

[15] Hou YJ , Chiba S, Halfmann P, et al SARS-CoV-2 D614G variant exhibits efficient replication ex vivo and transmission in vivo. Science 2020; 370:1464–8. 33184236 10.1126/science.abe8499 PMC7775736

[16] Dejnirattisai W , Huo J, Zhou D, et al SARS-CoV-2 omicron-B.1.1.529 leads to widespread escape from neutralizing antibody responses. Cell 2022; 185:467–84.e15. 35081335 10.1016/j.cell.2021.12.046 PMC8723827

[17] Cele S , Jackson L, Khoury DS, et al Omicron extensively but incompletely escapes Pfizer BNT162b2 neutralization. Nature 2022; 602:654–6. 35016196 10.1038/s41586-021-04387-1 PMC8866126

[18] Dejnirattisai W , Zhou D, Supasa P, et al Antibody evasion by the P.1 strain of SARS-CoV-2. Cell 2021; 184:2939–54.e9. 33852911 10.1016/j.cell.2021.03.055 PMC8008340

[19] Garcia-Beltran WF , Lam EC, St. Denis K, et al Multiple SARS-CoV-2 variants escape neutralization by vaccine-induced humoral immunity. Cell 2021; 184:2372–83.e9. 33743213 10.1016/j.cell.2021.03.013 PMC7953441

[20] Wang P , Nair MS, Liu L, et al Antibody resistance of SARS-CoV-2 variants B.1.351 and B.1.1.7. Nature 2021; 593:130–5. 33684923 10.1038/s41586-021-03398-2

[21] Wang SY , Juthani PV, Borges KA, et al Severe breakthrough COVID-19 cases in the SARS-CoV-2 delta (B.1.617.2) variant era. Lancet Microbe 2022; 3:e4–5. 34901896 10.1016/S2666-5247(21)00306-2 PMC8641954

[22] Pulliam JRC , van Schalkwyk C, Govender N, et al Increased risk of SARS-CoV-2 reinfection associated with emergence of Omicron in South Africa. Sciencce 2022; 376:eabn4947. 10.1126/science.abn4947 PMC8995029 35289632

[23] Wang L , Kaelber DC, Xu R, Berger NA. COVID-19 breakthrough infections, hospitalizations and mortality in fully vaccinated patients with hematologic malignancies: a clarion call for maintaining mitigation and ramping-up research. Blood Rev 2022; 54:100931. 35120771 10.1016/j.blre.2022.100931 PMC8802493

[24] UK HSA . COVID-19 vaccination: a guide to booster vaccination for individuals aged 18 years and over and those aged 16 years and over who are at risk: UK Government (Gov.uk), 2022.

[25] CDC . COVID-19 Vaccines Work. COVID-19 vaccines are effective. UK: Centers for Disease Control and Prevention, 2021.

[26] EMA . COVID-19 vaccines: key facts: COVID-19: European Medicines Agency, 2022.

[27] Sigel K , Pitts R, Crothers K. Lung malignancies in HIV infection. Semin Respir Crit Care Med 2016; 37:267–76. 26974303 10.1055/s-0036-1578803 PMC5140273

[28] Mellor MM , Bast AC, Jones NR, et al Risk of adverse coronavirus disease 2019 outcomes for people living with HIV. AIDS 2021; 35:F1–10. 33587448 10.1097/QAD.0000000000002836 PMC7924978

[29] Sheth AN , Althoff KN, Brooks JT. Influenza susceptibility, severity, and shedding in HIV-infected adults: a review of the literature. Clin Infect Dis 2011; 52:219–27. 21288848 10.1093/cid/ciq110 PMC4990828

[30] Tesoriero JM , Swain CE, Pierce JL, et al COVID-19 outcomes among persons living with or without diagnosed HIV infection in New York State. JAMA Netw Open 2021; 4:e2037069. 10.1001/jamanetworkopen.2020.37069 PMC7859843 33533933

[31] Pawlowski A , Jansson M, Sköld M, Rottenberg ME, Källenius G. Tuberculosis and HIV co-infection. PLoS Pathog 2012; 8:e1002464. 10.1371/journal.ppat.1002464 PMC3280977 22363214

[32] Mayer KH , Karp CL, Auwaerter PG, Mayer KH. Coinfection with HIV and tropical infectious diseases. II. Helminthic, fungal, bacterial, and viral pathogens. Clin Infect Dis 2007; 45:1214–20. 17918087 10.1086/522180

[33] Peluso MJ , Spinelli MA, Deveau T-M, et al Post-acute sequelae and adaptive immune responses in people living with HIV recovering from SARS-CoV-2 infection. MedRxiv 2022; 36:F7–16. 10.1097/QAD.0000000000003338 PMC9444925 35866847

[34] Crothers K , Huang L, Goulet JL, et al HIV Infection and risk for incident pulmonary diseases in the combination antiretroviral therapy era. Am J Respir Crit Care Med 2011; 183:388–95. 20851926 10.1164/rccm.201006-0836OC PMC3266024

[35] Munro APS , Janani L, Cornelius V, et al Safety and immunogenicity of seven COVID-19 vaccines as a third dose (booster) following two doses of ChAdOx1 nCov-19 or BNT162b2 in the UK (COV-BOOST): a blinded, multicentre, randomised, controlled, phase 2 trial. Lancet 2021; 398:2258–76. 34863358 10.1016/S0140-6736(21)02717-3 PMC8639161

[36] Flaxman A , Marchevsky NG, Jenkin D, et al Reactogenicity and immunogenicity after a late second dose or a third dose of ChAdOx1 nCoV-19 in the UK: a substudy of two randomised controlled trials (COV001 and COV002). Lancet 2021; 398:981–90. 34480858 10.1016/S0140-6736(21)01699-8 PMC8409975

[37] Bar-On YM , Goldberg Y, Mandel M, et al Protection of BNT162b2 vaccine booster against COVID-19 in Israel. N Engl J Med 2021; 385:1393–400. 34525275 10.1056/NEJMoa2114255 PMC8461568

[38] Yewdell JW . Antigenic drift: understanding COVID-19. Immunity 2021; 54:2681–7. 34910934 10.1016/j.immuni.2021.11.016 PMC8669911

[39] Barrett JR , Belij-Rammerstorfer S, Dold C, et al Phase 1/2 trial of SARS-CoV-2 vaccine ChAdOx1 nCoV-19 with a booster dose induces multifunctional antibody responses. Nat Med 2021; 27:279–88. 33335322 10.1038/s41591-020-01179-4

[40] He Q , Mao Q, An C, et al Heterologous prime-boost: breaking the protective immune response bottleneck of COVID-19 vaccine candidates. Emerg Microbes Infect 2021; 10:629–37. 33691606 10.1080/22221751.2021.1902245 PMC8009122

[41] Borobia AM , Carcas AJ, Pérez-Olmeda M, et al Immunogenicity and reactogenicity of BNT162b2 booster in ChAdOx1-S-primed participants (CombiVacS): a multicentre, open-label, randomised, controlled, phase 2 trial. Lancet 2021; 398:121–30. 34181880 10.1016/S0140-6736(21)01420-3 PMC8233007

[42] Francis AI , Ghany S, Gilkes T, Umakanthan S. Review of COVID-19 vaccine subtypes, efficacy and geographical distributions. Postgrad Med J 2021; 98:389–94. 37066438 10.1136/postgradmedj-2021-140654

[43] Global Initiative on Sharing Avian Influenza Data (GSAID) . Tracking of hCoV-19 variants. Webpage. Available at: https://gisaid.org/hcov19-variants. Accessed May 2022.

[44] Wang Z , Schmidt F, Weisblum Y, et al mRNA vaccine-elicited antibodies to SARS-CoV-2 and circulating variants. Nature 2021; 592:616–22. 33567448 10.1038/s41586-021-03324-6 PMC8503938

[45] Ogbe A , Kronsteiner B, Skelly DT, et al T cell assays differentiate clinical and subclinical SARS-CoV-2 infections from cross-reactive antiviral responses. Nat Commun 2021; 12:2055. 33824342 10.1038/s41467-021-21856-3 PMC8024333

[46] Ewer KJ , Barrett JR, Belij-Rammerstorfer S, et al T cell and antibody responses induced by a single dose of ChAdOx1 nCoV-19 (AZD1222) vaccine in a phase 1/2 clinical trial. Nat Med 2021; 27:270–8. 33335323 10.1038/s41591-020-01194-5

[47] Liu X , Shaw RH, Stuart ASV, et al Safety and immunogenicity of heterologous versus homologous prime-boost schedules with an adenoviral vectored and mRNA COVID-19 vaccine (Com-COV): a single-blind, randomised, non-inferiority trial. Lancet 2021; 398:856–69. 34370971 10.1016/S0140-6736(21)01694-9 PMC8346248

[48] Mudd PA , Minervina AA, Pogorelyy MV, et al SARS-CoV-2 mRNA vaccination elicits a robust and persistent T follicular helper cell response in humans. Cell 2022; 185:603–13.e15. 35026152 10.1016/j.cell.2021.12.026 PMC8695127

[49] Pardi N , Hogan MJ, Naradikian MS, et al Nucleoside-modified mRNA vaccines induce potent T follicular helper and germinal center B cell responses. J Exp Med 2018; 215:1571–88. 29739835 10.1084/jem.20171450 PMC5987916

[50] Nielsen CM , Ogbe A, Pedroza-Pacheco I, et al Protein/AS01B vaccination elicits stronger, more Th2-skewed antigen-specific human T follicular helper cell responses than heterologous viral vectors. Cell Rep Med 2021; 2:100207. 33763653 10.1016/j.xcrm.2021.100207 PMC7974546

