# Supplementary material for: Booster Vaccination Against SARS-CoV-2 Induces Potent Immune Responses in People With Human Immunodeficiency Virus
Source: Clin Infect Dis. 2022 Oct 5;76(2):201–9. doi: 10.1093/cid/ciac796 (PMC9619587; doi:10.1093/cid/ciac796)
Supplement: ciac796_Supplementary_Data [file ciac796_supplementary_data.docx]

**Supplementary Material**

**Tables**

| **Participant** | **Days post boost when samples** | **Booster type** |
| --- | --- | --- |
| Donor_1 | 1 | Pfizer |
| Donor_2 | 5 | Pfizer |
| Donor_3 | 5 | Pfizer |
| Donor_4 | 5 | Pfizer |
| Donor_5 | 6 | Pfizer |
| Donor_6 | 6 | Pfizer |
| Donor_7 | 11 | Pfizer |
| Donor_8 | 16 | Pfizer |
| Donor_9 | 18 | Pfizer |
| Donor_10 | 18 | Pfizer |
| Donor_11 | 19 | Pfizer |
| Donor_12 | 20 | Pfizer |
| Donor_13 | 23 | Pfizer |
| Donor_14 | 24 | Pfizer |
| Donor_15 | 27 | Pfizer |
| Donor_16 | 27 | Pfizer |
| Donor_17 | 28 | Pfizer |
| Donor_18 | 28 | Pfizer |
| Donor_19 | 28 | Pfizer |
| Donor_20 | 29 | Pfizer |
| Donor_21 | 30 | Pfizer |
| Donor_22 | 32 | Pfizer |
| Donor_23 | 32 | Pfizer |
| Donor_24 | 35 | Pfizer |
| Donor_25 | 35 | Pfizer |
| Donor_26 | 35 | Pfizer |
| Donor_27 | 36 | Pfizer |
| Donor_28 | 36 | Pfizer |
| Donor_29 | 37 | Pfizer |
| Donor_30 | 38 | Pfizer |
| Donor_31 | 39 | Pfizer |
| Donor_32 | 39 | Pfizer |
| Donor_33 | 40 | Pfizer |
| Donor_34 | 41 | Pfizer |
| Donor_35 | 41 | Pfizer |
| Donor_36 | 42 | Pfizer |
| Donor_37 | 42 | Pfizer |
| Donor_38 | 47 | Pfizer |
| Donor_39 | 47 | Pfizer |
| Donor_40 | 52 | Pfizer |
| Donor_41 | 54 | Pfizer |
| Donor_42 | 59 | Pfizer |
| Donor_43 | 62 | Pfizer |
| Donor_44 | 115 | Moderna |

**Supplementary Table 1**

|  | **Fluorochrome** | **Marker** | **Clone** | **Volume/50ul** | **Catalogue No** | **Company** |
| --- | --- | --- | --- | --- | --- | --- |
| CTV Proliferation Assay |  | L/D NIR |  |  | L24976 | Invitrogen |
|  |  | CTV |  |  | C34557 | Invitrogen |
|  | FITC | CD3 | UCHT1 | 1 | 300440 | Biolegend |
|  | APC | CD4 | RPA-T4 | 0.25 | 300537 | Biolegend |
|  | PE CY7 | CD8 | RPA-T8 | 0.25 | 301012 | Biolegend |

**Supplementary Table 2**

|  | **Fluorochrome** | **Marker** | **Clone** | **Volume/50ul** | **Catalogue No** | **Company** |
| --- | --- | --- | --- | --- | --- | --- |
| AIM assay | APC | CXCR3 | 1C6/CXCR3 | 5 | 550967 | BD |
|  | BB515 | CXCR5 | RF8B2 | 1 | 564624 | BD |
|  | APC CY7 | CCR6 | G034E3 | 2 | 353432 | Biolegend |
|  | BV421 | PD1 | EH12.2H7 | 1 | 329920 | Biolegend |
|  | BV510 | CD19 | HBI19 | 0.25 | 302242 | Biolegend |
|  | BV510 | CD14 | M5E2 | 0.25 | 301842 | Biolegend |
|  | PE CF 594 | CD137 | 4B4-1 | 1 | 309826 | Biolegend |
|  | BV650 | CD69 | FN50 | 0.5 | 310934 | Biolegend |
|  | BV570 | CD4 | SK3 | 1 | 300534 | Biolegend |
|  | PERCP EFLUOR 710 | CD39 | eBioA1 | 1.5 | 46-0399-42 | Invitrogen |
|  | PE | CD134 (OX40) | L106 | 2 | 340420 | BD |
|  |  | L/D AQUA |  |  | L34966 | Invitrogen |
|  | PECY7 | CD25 | 2A3 | 1 | 335824 | BD |
|  | APCR700 | CD8 | RPA-T8 | 0.25 | 565165 | BD |
|  | BV605 | CD3 | UCHT1 | 0.5 | 300460 | Biolegend |

**Supplementary Table 3**

**Supplementary Figure 1 - Longitudinal antibody responses in PWH. A)** complete longitudinal analysis of total SARS-CoV-2 antibody IgG titres in volunteers with day 365 pre and post boost samples. **B)** longitudinal analysis for all HIV+ volunteers measured using In-house ELISA. Kinetics of ACE-2 binding antibodies for **C)** SARS-CoV-2. **D)** Alpha, **E)** Beta, **F)** Gamma in plasma of volunteers with day 365 pre and post boost. Statistical test was done by Wilcoxon matched pair sign ranked test. Where indicated * = <0.05, ** = <0.01, *** = < 0.001 and **** = <0.000. ‘Pre-B’ and ‘Post-B’ refer to pre-third dose and post-third dose. Dotted lines in indicate cut off points determined for each SARS-CoV-2 spike antigen based on pre-pandemic sera + 3X SD. n = 9 for HIV+ volunteers. Error bars represent median and interquartile range.

Comp-Pacific Blue

Comp-Pacific Blue

Comp-Pacific Blue

Comp-Alexa Fluor700

Comp-Alexa Fluor700

Comp-Alexa Fluor700

ComppPE-Cy7

ComppPE-Cy7

ComppPE-Cy7

**Supplementary Figure 2 - Flow cytometer gating strategy and T cell responses to control antigens across HIV+ participant timepoints.** Gating strategy for **A**) AIM and control responses for AIM assay showing CD4+ and CD8+ T cell response to SEB (**B and C**) and CMVPP65 peptide pool (**D and E**) at days 182 and 365. Gating strategy for **F**) proliferation assay and control responses for proliferation assay showing CD4+ and CD8+ T cell response to PHA (**G and H**) and CMVPP65 peptide pool (**I and J**) at baseline, and days 182 and 365. Statistical test in B – D was done by Mann Whitney T test. Statistical test in G – J was done by Wilcoxon matched pair sign ranked test. Fold change was calculated by dividing the medians of the two timepoints of interest. Where indicated * = <0.05, ** = <0.01, *** = < 0.001 and **** = <0.000. Dotted lines in indicate cut off points determined based on DMSO controls + 3X SD. n = 24 – 40 for AIM assay and 41 – 52 for proliferation assay. Error bars represent median and interquartile range.

**Supplementary Figure 3 - Longitudinal analysis of proliferative response following third dose of COVID-19 vaccines at all timepoints**. Proliferative T cell response in CD4+ **A)** SARS-CoV-2 S1, **B)** SARS-CoV-2 S2 and CD8+ **C)** SARS-CoV-2 S1, **D)** SARS-CoV-2 S2 in all participants and CD4+ **E)** SARS-CoV-2 S1, **F)** SARS-CoV-2 S2 and CD8+ **G)** SARS-CoV-2 S1, **H)** SARS-CoV-2 S2 in participants with day 365 pre and post boost timepoints. Statistical test was done by Wilcoxon matched pair sign ranked test. Where indicated * = <0.05, ** = <0.01, *** = < 0.001 and **** = <0.000. ‘Pre-B’ and ‘Post-B’ refer to pre-third dose and post-third dose Dotted lines in indicate cut off points determined based on DMSO controls + 3X SD. n = 24 – 40 for AIM assay and 41 – 54 for proliferation assay. Error bars represent median and interquartile range.

**Supplementary Figure 4 - Immune response to VOCs following COVID-19 booster vaccination**. Antibody immune responses to **A)** Alpha, **B)** Beta and **C)** Gamma and Proliferative T cell response to Beta, Gamma and Delta VOCs in CD4+ **D-F)** SARS-CoV-2 S1, **G-I)** SARS-CoV-2 S2 and CD8+ **J-L)** SARS-CoV-2 S1, **M-O)** SARS-CoV-2 S2. Comparison of two timepoints within the same group was done by Wilcoxon matched pair sign ranked test. Where indicated * = <0.05, ** = <0.01, *** = < 0.001 and **** = <0.000. Dotted lines in indicate cut off points determined for each SARS-CoV-2 spike antigen based on pre-pandemic sera + 3X SD for antibody responses and cut off points determined based on DMSO controls + 3X SD for proliferative responses. n = 37 - 40 for antibody analysis and n = 24 or proliferation assay. Error bars represent median and interquartile range.

**Supplementary Figure 5 - Omicron variant responses are moderately boosted in response to COVID-19 booster vaccines**. antibody response in participants with Pre and post booster vaccine timepoints showing **A)** Total IgG and **B)** ACE-2 inhibition and proliferative T cell response in **C)** CD4+ and **D)** CD8+ T cells to Omicron spike peptide pools. Comparison of two timepoints within the same group was done by Wilcoxon matched pair sign ranked test. Where indicated * = <0.05, ** = <0.01, *** = < 0.001 and **** = <0.000. ‘Pre-B’ and ‘Post-B’ refer to pre-third dose and post-third dose. Dotted lines in indicate cut off points determined for each SARS-CoV-2 spike antigen based on pre-pandemic sera + 3X SD for antibody responses and cut off points determined based on DMSO controls + 3X SD for proliferative responses. n = 9 for both antibody analysis and proliferation assay. Error bars represent median and interquartile range.

**Detailed Methods for Laboratory Assays**

**SARS CoV-2 spike IgG ELISA**

Humoral responses at baseline and following vaccination were assessed using a standardised total IgG ELISA against trimeric SARS CoV-2 spike protein. ELISA plates were coated with 2 μg/mL of full-length trimerised SARS-CoV-2 spike glycoprotein and stored at 4°C overnight for at least 16 hours. After coating, plates were washed 6 times with PBS/0.05%Tween and blocked with casein for 1h at room temperature (RT). Thawed samples were treated with 10% Triton X-100 for 1 h at RT and subsequently diluted in casein and plated in triplicate for incubation for 2h at RT alongside two internal positive controls (controls 1 and 2) to measure plate to plate variation. Control 1 was a dilution of convalescent plasma sample and control 2 was a research reagent for anti-SARS-CoV-2 Ab (code 20/130 supplied by National Institute for Biological Standards and Control (NIBSC)). The standard pool was used in a two-fold serial dilution to produce ten standard points that were assigned arbitrary ELISA units (EUs). Goat anti-human IgG (γ-chain specific) conjugated to alkaline phosphatase was used as secondary antibody and plates were developed by adding 4-nitrophenyl phosphate in diethanolamine substrate buffer. An ELx808 microplate reader (BioTek Instruments) was used to provide optical density measurement of the plates at 405mm. Standardised EUs were determined from a single dilution of each sample against the standard curve which was plotted using the 4-Parameter logistic model (Gen5 v3.09, BioTek). Each assay plate consisted of samples and controls plated in triplicate, with ten standard points in duplicate and four blank wells.

**Mesoscale Discovery (MSD) binding assays**

IgG responses to SARS-CoV-2 variant spike antigens including Wuhan strain, Alpha, Beta, Gamma, Delta, Omicron were measured using a multiplexed V-PLEX COVID-19 Coronavirus Panel 23 Kit (K15570U-2) from Meso Scale Diagnostics, Rockville, MD USA. A MULTI-SPOT^®^ 96-well, 8 spot plate was coated with 8 variant spike trimers and bovine serum albumin. Multiplex MSD assays were performed as per the instructions of the manufacturer. To measure IgG antibodies, 96-well plates were blocked with MSD Blocker A for 30 minutes. Following washing with washing buffer, samples diluted 1: 10,000 in diluent buffer, or MSD reference standard 1 (Lot A0080286) or undiluted internal MSD controls (Lot A00C0771, A00C0772, A00C0773) were added to the wells. After 2-hour incubation and a washing step, detection antibody (MSD SULFO-TAG™ Anti-Human IgG Antibody, 1/200) was added. Following washing, MSD GOLD™ Read Buffer B was added and plates were read using a MESO^®^ SECTOR S 600 Reader. The standard curve was established by fitting the signals from the standard using a 4-parameter logistic model. Concentrations of samples were determined from the electrochemiluminescence signals by back-fitting to the standard curve and multiplied by the dilution factor. Concentrations are expressed in Arbitrary Units/ml (AU/ml). Cut-offs were determined for SARS-CoV-2 Wuhan spike based on the concentrations measured in 103 pre-pandemic sera + 3 Standard Deviations. Cut-off for Wuhan S: 1160 AU/ml.

**T cell proliferation assay**

Cryopreserved PBMCs were thawed and washed twice with 1mL of PBS followed by labelling with CTV at a final concentration of 2·5μM for 10 min at room temperature. The labelling reaction was quenched with 4mL of fetal bovine serum (FBS) at 4°C and cells were resuspended in RPMI medium supplemented with 10% human blood group type AB serum (Sigma), 1mM penicillin-streptomycin solution, and 2 mM L-glutamine solution, and subsequently plated in a 96-well round bottom plate at a plating density of 0·25 × 10^6^ cells per well. Cells were stimulated with peptide pools (15-mers overlapping by 11) spanning SARS-CoV-2 spike (S1 and S2), SARS-CoV-2 variants of concern (Beta, Gamma and Delta and Omicron) at a final concentration of 1μg/mL per peptide. For antigenic control, class 1 and 2 optimal peptides for FEC-T (flu, EBV, CMV, and tetanus) were pooled at a final concentration of 1μg/mL per peptide. Media, containing 0·1% dimethyl sulfoxide (DMSO; Sigma) representing DMSO content in peptide pools, was used as a negative control and 2μg/mL phytohaemagglutinin L (Sigma) was used as positive control. Cells were then incubated at 37°C, with 5% CO_2_ for 7 days, with a change of media on day 4. At the end of the incubation period, cells were stained using the antibody panel in **Supplementary Table 2**. All samples were acquired using a BD Fortessa X20 (BD Bioscience, San Jose, CA, USA) or MACSQuant x10 (Miltenyi Biotec, Bergisch Gladbach, Germany) and the gating strategy is shown in **Supplementary Figure 2F**. Responses above 1% were considered true positive based mean of DMSO controls + 3x SD. Specificity of the assay has been previous reported in (58). All datapoints presented represent a single participant and are presented as background subtracted data.

**AIM Assay**

Cryopreserved PBMCs were thawed in R10 (RPMI + 10% FCS, 1% Pen/strep and 1% L-glutamine. Cells were washed, counted, and rested for 3 hours in IMDM-10 (Iscove’s Modified Dulbecco’s Medium - Sigma, I3390 + 10% Human AB serum, 1% Pen/strep and 1% L-glutamine) and 1ul/ml of benzonase nucleases (70746-3, Merck). Following rest, cells were plated at 1-2 x10^6^ cells/well in a 96 well round bottom plate and incubated for 24 hours at 37ºC and 5% CO2. After stimulation cells were stained with the anti-human antibodies contained in **Supplementary Table 3** followed by 4% PFA fixation and acquisation on a BD LSR II flow cytometer. The data was analysed using FlowJo v10. Antigen-specific CD4+ and CD8+ T cells were gated using the Boolean OR gating strategy described by Nielsen and Ogbe et al, 2020 (59) and shown in **Supplementary Figure 2a (**for CD4 T cells: all double positive CD25+ CD134(OX40)+ or CD25+ CD137+ or CD25+ CD69+ were considered AIM+; for CD8+ T cells: all double positive CD25+ CD137+ or CD25+ CD69+ were considered AIM+). Chemokine receptors CCR6 and CXCR3 were used as an unbiased way of analysing T cell skewness independent of cytokine kinetics.

**MSD ACE-2 inhibition assay**

A multiplexed MSD immunoassay (MSD, Rockville, MD) was used to measure the ability of human sera to inhibit ACE-2 binding to SARS-CoV-2 spike (B, B.1, B.1.1.7, B.1.351 or P.1, B.1.617, B.1.1.59). A MULTI-SPOT® 96-well, 10 Spot Plate (Plate 7) was coated with eight spike proteins to SARS-CoV-2 and circulating variants of concern (B, B.1, B.1.1.7, B.1.351 or P.1, B.1.617, B.1.1.59. Multiplex MSD Assays were performed as per manufacturer’s instructions. To measure ACE-2 inhibition, 96-well plates were blocked with MSD blocker for 30 minutes. Plates were then washed in MSD washing buffer, and samples were diluted 1:10 and 1:100 in diluent buffer. Importantly, an ACE-2 calibration curve which consisting of a monoclonal antibody with equivalent activity against spike variants was used to interpolate results as arbitrary units. This was done for the ancestral strain and all other VOCs except for Omicron. To report antibody inhibition of ACE-2 binding by Omicron compared to other VOCs, % inhibition was calculated according to the manufacturer’s instructions. Furthermore, internal controls and the WHO international standard were added to each plate. After 1-hour incubation recombinant human ACE2-SULFO-TAG™ was added to all wells. After a further 1-hour plates were washed and MSD GOLD™ Read Buffer B was added, plates were then immediately read using a MESO® SECTOR S 600 Reader. Where antibody levels were below detection limit of the assay, the minimum detection limit of 0.1AU/ml was used.
